# The Value of a Man

**Published:** 1913-11-01

**Authors:** 


### The Value of a Man.

Some little time ago an exposition was published in these pages (The Hospital, April 19, 1913, p. 57) of a new mathematical scheme from America for assessing the value of a man or any part of him. This contained elaborate tables for calculating such matters as proper compensation for injuries, death, and so forth, due to causes for which some other person or corporation was liable to make a monetary atonement to the individual concerned. A different estimation of the Value of a Man comes from Germany, where a scientific author has been computing the intrinsic value of a human being at current prices for the various components into which he can be dismembered. For a man (or woman) weighing 150 lbs., or 10 stone 10 lbs., the cash equivalent is said to be £1 11s. 3d. This value is assessed on the phosphorus, lime, iron, sulphur, albumin, and other constituents of the body. The fat, for instance, is worked out at about ten shillings and five pence: obviously this must vary a good deal according to the condition of the individual, and the corpulent may derive comfort by observing how valuable, compared with other tissues, this ingredient of the human frame appears to be. Of iron, it is stated, there is hardly enough to make a small nail an inch in length; but of lime there is plenty to whitewash a fair-sized chicken-house. The phosphorus would be sufficient to tip 2,200 matches, and there is also enough magnesium to make a fine flashlight. The body (of an individual of the weight specified) contains as much albumin as one hundred eggs. A teaspoonful of sugar and a pinch of salt are also said to be present: the former of these we should have thought was a low estimate. There must be a good many people whose gold-filled teeth represent a greater intrinsic worth than the whole of the rest of their bodies!

